# The association between benign and malignant prostatic hyperplastic diseases and blood and urine biomarkers: A Mendelian randomization study

**DOI:** 10.1097/MD.0000000000041723

**Published:** 2025-03-07

**Authors:** Ze-sen Lin, Ze-chao Zhang, Gui-lan Ning, Shu-ping Huang, Di-kuan Yang, Bao-zhong Wu, Zhi-xiong Hu, Min Zhu, Ming-jian Liu

**Affiliations:** a Department of Urology, The Second People’s Hospital of Zhaoqing, Zhaoqing, China; b Department of Traditional Chinese Medicine Surgery, Ruikang Hospital Affiliated to Guangxi University of Chinese Medicine, Nanning, China; c Department of Graduate School, Guangxi University of Chinese Medicine, Nanning, China.

**Keywords:** benign prostatic hyperplasia, blood and urine biomarkers, Mendelian randomization, prostate cancer

## Abstract

The identification of suitable biomarkers holds significant clinical importance for the early detection of benign prostatic hyperplasia (BPH) and prostate cancer (PCa). This study aimed to conduct a comprehensive analysis of the relationship between BPH and blood and urine biomarkers (BUB), as well as PCa and BUB. Candidate single nucleotide polymorphisms associated with BPH or PCa were derived from recent genome-wide association studies. The UK Biobank cohort comprised 363,228 individuals with BUB test data, enabling the calculation of a polygenic risk score for BPH or PCa. Bidirectional 2-sample Mendelian randomization was employed to assess the potential causal association between candidate BUB and BPH, as well as between BUB and PCa. In this study, a notable correlation was observed between BPH and gamma-glutamyl transferase (*r* = 1.061658447, *P* = .028697428). PCa exhibited significant associations with insulin-like growth factor 1 (*r* = 1.119051258, *P* = .004101067), Lipoprotein A (*r* = 1.120348291, *P* = .038093372), total protein (*r* = 0.87643517, *P* = .01657563), and non-albumin protein (*r* = 0.905333153, *P* = .03103913). The findings offer valuable insights into the significance of BUB in the timely identification and management of BPH and PCa.

## 1. Introduction

Benign prostatic hyperplasia (BPH) is characterized by a nonmalignant enlargement of the prostate gland, which arises from the unregulated proliferation of epithelial and fibromuscular tissues within the transition zone and the surrounding areas of the urethra. The prevalence of histologically diagnosed BPH in men has risen significantly from 41% to 90% annually, with BPH changes observed in 50% of men aged 51 to 60 years. The most prevalent issues associated with BPH include diminished urinary flow, increased frequency of urination, difficulty initiating urination, post-void dribbling (lower urinary tract symptoms during the night), all of which significantly impact the quality of life of affected individuals.^[1]^ The prevalence of individuals seeking medical intervention for lower urinary tract symptoms associated with BPH has been increasing, resulting in a corresponding rise in healthcare costs.^[2]^ Diagnosing benign prostatic hyperplasia (BPH) involves evaluating clinical symptoms, predominantly lower urinary tract symptoms, the International Prostate Symptom Score, urinary flow rate, digital rectal examination, ultrasound, and prostate-specific antigen (PSA), among others. A PSA level below 4 ng/mL is typically regarded as normal; however, various factors can affect PSA levels, which can reduce its specificity in diagnosing BPH.^[3,4]^ It is crucial to note that while PSA levels are significant, they should not be utilized solely for the diagnosis of BPH. The acquisition of more specific indicators for BPH is of considerable importance for its diagnosis and treatment. In males aged 65 years and older, the prevalence of prostate cancer (PCa) is approximately 60%, making it the second most frequently diagnosed cancer among men.^[5]^ Men with PCa are typically diagnosed through prostate biopsy, analysis of PSA levels, digital rectal examination, magnetic resonance imaging, or physical examination.^[6]^ Cancer remains a leading cause of death globally, necessitating the identification of prognostic biomarkers to guide treatment decisions.^[7,8]^ Individuals exhibiting PSA levels ranging from 4 to 10 ng/mL have an estimated probability of approximately 25% for the development of prostate cancer. Conversely, when PSA levels surpass 10 ng/mL, the likelihood of developing prostate cancer increases to over 50%.^[9]^ In addition to ultrasound, the conventional diagnostic techniques for BPH are characterized by a lack of convenient, specific, and highly accurate examination conditions. Ultrasound examinations are subject to equipment limitations and may be impractical in remote or underserved areas, as well as when conducting screenings on a large scale. In addition to prostate biopsy, other examinations, such as PSA and magnetic resonance imaging, are associated with drawbacks related to convenience and specificity. The implementation of a standardized, convenient, and timely testing procedure holds significant clinical importance in the context of screening, early intervention, and assessment of BPH and PCa.

Mendelian randomization (MR) is an epidemiological approach that leverages genetic variants as instrumental variables to assess the causal relationships between exposures and outcomes.^[10]^ The random distribution of single nucleotide polymorphisms (SNPs) helps mitigate confounding factors. Since genetic variations cannot be influenced by subsequent outcome traits, this reduces the potential for reverse causality bias.^[11]^ High-throughput metabolomics advancements allow the simultaneous measurement of blood and urine biomarkers (BUB). The association between BUB and SNPs was extensively studied in a genome-wide association study (GWAS) involving 363,228 samples and examining 35 serum and urine biomarkers.^[12]^ Both BPH and PCa exhibit a high prevalence. Large-scale multi-data analysis is beneficial for enhancing comprehension of diseases and refining treatment approaches. In this study, we utilized the most extensive human genome dataset available to date to apply the MR approach. We aimed to discern the inherent relationship between BPH and BUB, as well as between PCa and BUB.

## 2. Materials and methods

### 2.1. Research design description

**Figure 1.**
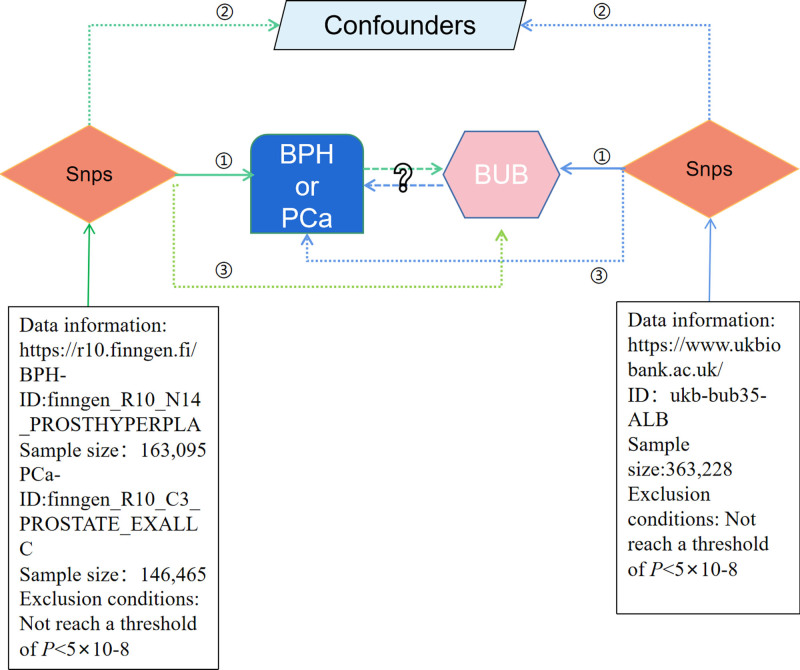
Flow chart. Relevance: genes are associated with the exposure. Independence: genes are not associated with any confounder of the exposure-outcome. Exclusion restriction: genes do not affect outcomes except through their potential effect on the exposure.

Figure 1 outlines the key steps of the bidirectional MR study investigating the interplay between BUB and BPH or PCa. This study involves 2 MR analyses, using summary statistical data from GWAS, to unveil potential associations between BUB and BPH or PCa. In the forward MR analysis, BUB is treated as the exposure variable and BPH or PCa as the outcome. In the reverse MR analysis, BPH or PCa is considered the exposure variable and BUB the outcome. The fundamental MR assumptions underpinning the study are illustrated in Figure 1 and Table 1. Given that this study relies on publicly available data, ethical approval is not required. MR Analysis was performed with reference to the STROBE-MR checklist.^[13]^

**Table 1:** Exposure and outcome assignment for MR.

MR	Exposure variable	Outcome
BPH forward MR	BUB	BPH
BPH reverse MR	BPH	BUB
PCa forward MR	BUB	PCa
PCa reverse MR	PCa	BUB

BPH = benign prostatic hyperplasia, BUB = blood and urine biomarkers, MR = Mendelian randomization, PCa = prostate cancer.

### 2.2. MR tool variable selection

The instrumental variables (IVs) used in the MR analysis were derived from 2 separate GWAS summary datasets. A genome-wide significance threshold of *P* < 5 × 10^−8^ was applied initially.^[14]^ Subsequently, SNP independence was assessed using pairwise linkage disequilibrium. SNPs with an *r*^2^ > 0.001 within a 10,000 kb window were excluded to effectively account for associations involving multiple SNPs and those with higher *P* values.^[15]^ Linkage disequilibrium refers to the nonrandom association of alleles at different loci. Essentially, if 2 genes are inherited together, some degree of linkage will be observed. The parameter *r*^2^ ranges from 0 to 1, where *r*^2^ = 1 represents complete linkage disequilibrium and *r*^2^ = 0 indicates complete linkage equilibrium, reflecting a random distribution of the 2 SNPs. The length of the linkage disequilibrium region is measured in kilobases (kb). To exclude SNPs with *r*^2^ values >0.001 within a 10,000 kb window, a threshold of *r*^2^ = 0.001 and a 10,000 kb window were applied. Additionally, *F* statistics were calculated to assess the strength of each individual SNP, with SNPs having an *F* statistic >10 considered sufficiently strong to minimize potential bias.^[16]^

### 2.3. BPH and PCa data source and tool variable selection

The BPH and PCa data originates from the FINNGEN pipeline version 10 (available at: https://r10.finngen.fi/). This dataset encompasses a population primarily diagnosed with BPH or PCa. The GWAS data is leveraged to identify SNPs associated with BPH or PCa, which are subsequently selected as IVs (Table 2).

**Table 2:** Detailed information of included data sources.

Traits	Sample size	Year	Web source
Blood and urine biomarkers	363,228	2016	https://biobank.ndph.ox.ac.uk/
Hyperplasia of prostate	163,095	2023	https://r10.finngen.fi/
Prostate cancer	146,465	2023	https://r10.finngen.fi/

### 2.4. BUB data source and tool variable selection

A summary-level GWAS dataset encompassing 249 CM was acquired from the UK Biobank (available at: https://biobank.ndph.ox.ac.uk; Table 2).

### 2.5. MR statistical analysis

SNPs associated with both BUB and BPH, as well as BUB and PCa, were utilized in the subsequent forward and reverse Mendelian randomization (MR) analyses. The random-effects inverse variance weighting (IVW) method, which aligns with the fundamental MR assumptions, served as the primary statistical approach for estimating potential bidirectional causal relationships between BUB and BPH, as well as BUB and PCa.^[17]^ When multiple IVs are available, the inverse variance weighting (IVW) method is considered the most reliable, as it incorporates variant-specific effects and accounts for heterogeneity in causal estimation. Additionally, the IVW method includes sensitivity analyses such as the simple mode, weighted mode, weighted median, and MR Egger regression, which help to evaluate the robustness of the research findings.^[18]^ If IVs influence outcomes through alternative pathways, suggesting potential pleiotropy, causal estimates derived from IVW may be biased. To evaluate pleiotropy, we applied the MR-Egger method. A *P* value >.05 in MR-Egger indicates no evidence of directional pleiotropy. Heterogeneity testing was performed using MR heterogeneity to detect heterogeneity induced by SNPs. In cases where heterogeneity was observed, a random-effects model was adopted; otherwise, a fixed-effects model was used by default. To assess the collective influence of individual SNPs, we sequentially excluded each SNP from the MR analysis.^[19]^ The Twosamplemr (v.0.5.6) within the R package (v.4.3.0) facilitated major statistical analysis and graphical representation.^[20]^ Odds ratio (OR) and the accompanying 95% confidence interval (CI) gauged the extent of risk alteration for each additional standard deviation of exposure factors. Statistical significance was set at *P* < .05.^[21]^ All data in this study were obtained from the GWAS database, and there were no missing data. Our execution code is available in the Supplementary file 1, Supplemental Digital Content, http://links.lww.com/MD/O456 (Code).

## 3. Results

### 3.1. BPH forward MR

**Figure 2.**
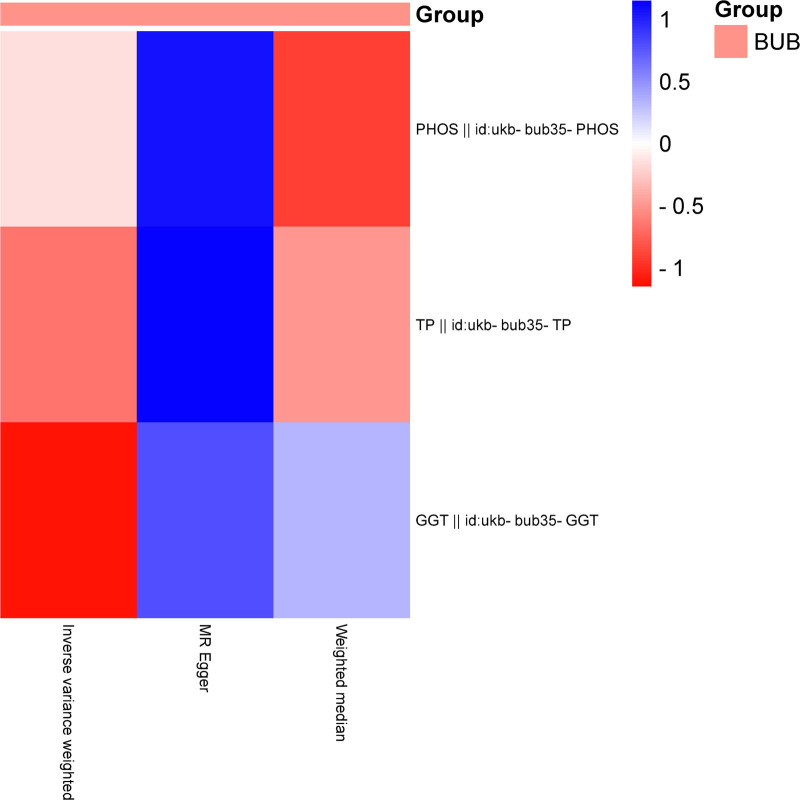
Heatmap illustrating significant correlations in forward MR. The figure showcases varying *P* values in distinct blocks, color-coded from red to blue denoting ascending *P* values. BUB = blood and urine biomarkers, MR = Mendelian randomization.

**Figure 3.**
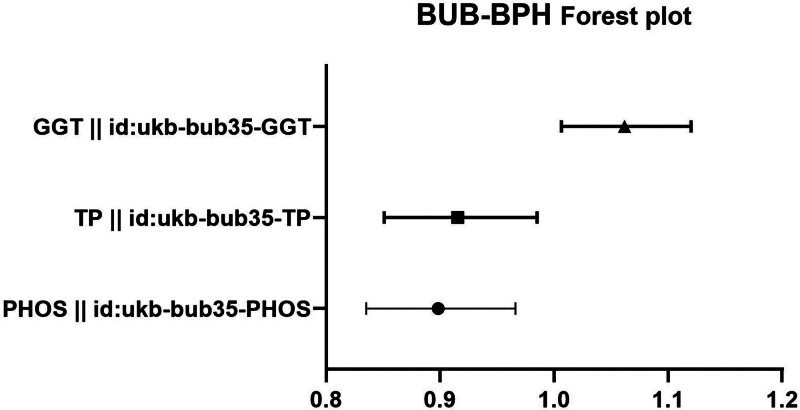
Forest plot depicting significant correlations in forward MR. The Y-axis represents diverse circulating metabolites, while the X-axis indicates OR values and corresponding 95% CIs. Various shapes in the graph symbolize distinct metabolite ORs, with the horizontal line denoting the range of the 95% CI. BPH = benign prostatic hyperplasia, BUB = blood and urine biomarkers, CI = confidence interval, MR = Mendelian randomization, OR = odds ratio.

The IVW analysis reveals a significant genetic correlation (*P* < .05) between 3 BUB and BPH (Table 3, Figs. 2 and 3). The MR analysis further indicated that gamma glutamyltransferase (GGT) genetic predisposition to BUB was associated with an increased risk of BPH, with an OR of 1.061658447 (95% CI: 1.006244869–1.120123634). The MR analysis further indicated that PHOS and total protein (TP) genetic predisposition to BUB was associated with an decreased risk of BPH, with an OR of 0.898347143402271 (95% CI: 0.835284610170002–0.966170788056025) and an OR of 0.915526028960424 (95% CI: 0.850927466734902–0.985028621675896). No substantial evidence of horizontal pleiotropy among SNPs is observed (Table 4, *P* > .05). Through MR Egger outcomes, only 1 (ukb-bub35-GGT) substantial heterogeneity is not detected in relation to the association (Table 5).

**Table 3:** Forward MR IVW of BUB and BPH.

ID exposure	ID outcome	Method	NSNP	*P* value	OR
ukb-bub35-PHOS	finngen_R10_N14_PROSTHYPERPLA	Inverse variance weighted	157	.003892414	0.898347143
ukb-bub35-TP	finngen_R10_N14_PROSTHYPERPLA	Inverse variance weighted	216	.018075926	0.915526029
ukb-bub35-GGT	finngen_R10_N14_PROSTHYPERPLA	Inverse variance weighted	259	.028697428	1.061658447

BPH = benign prostatic hyperplasia, BUB = blood and urine biomarkers, IVW = Inverse variance weighted, MR = Mendelian randomization.

**Table 4:** Forward MR horizontal pleiotropy of BUB and BPH.

ID exposure	ID outcome	Egger_intercept	SE	*P* value
ukb-bub35-PHOS	finngen_R10_N14_PROSTHYPERPLA	0.002148596	0.001906528	.261497803
ukb-bub35-TP	finngen_R10_N14_PROSTHYPERPLA	−0.001189486	0.002128348	.576829662
ukb-bub35-GGT	finngen_R10_N14_PROSTHYPERPLA	−0.00088059	0.001628322	.589116633

BPH = benign prostatic hyperplasia, BUB = blood and urine biomarkers, MR = Mendelian randomization.

**Table 5:** Forward MR heterogeneity of BUB and BPH.

ID exposure	ID outcome	Method	*Q*_pval
ukb-bub35-PHOS	finngen_R10_N14_PROSTHYPERPLA	MR Egger	0.000273043
ukb-bub35-TP	finngen_R10_N14_PROSTHYPERPLA	MR Egger	0.003220687
ukb-bub35-GGT	finngen_R10_N14_PROSTHYPERPLA	MR Egger	4.40E−06

BPH = benign prostatic hyperplasia, BUB = blood and urine biomarkers, MR = Mendelian randomization.

The findings indicate that the identified genetic association between BUB and BPH is not attributable to outliers or particular genetic variants. Furthermore, the MR-PRESSO global test did not reveal any outliers that could have significantly impacted the results. Collectively, these analyses offer robust evidence supporting a causal relationship between BUB and BPH, as evidenced by the genetic correlation established through the IVW method.

### 3.2. BPH reverse MR

No significant BUB was observed during the period of BPH exposure in the reverse MR Analysis.

### 3.3. PCa forward MR

**Figure 4.**
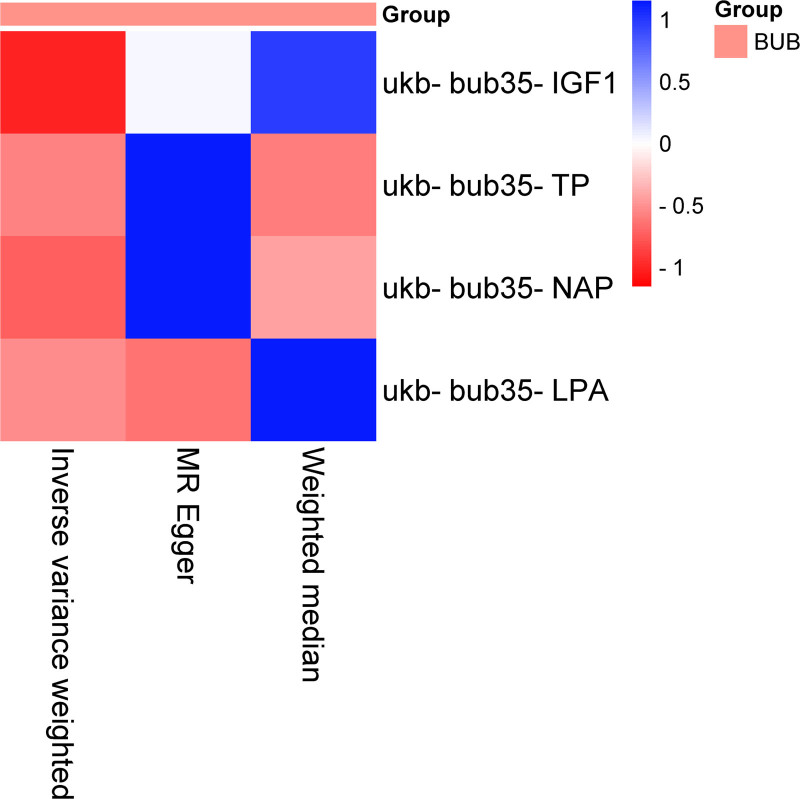
Heatmap illustrating significant correlations in forward MR. The figure showcases varying *P* values in distinct blocks, color-coded from red to blue denoting ascending *P* values. BUB = blood and urine biomarkers, MR = Mendelian randomization.

**Figure 5.**
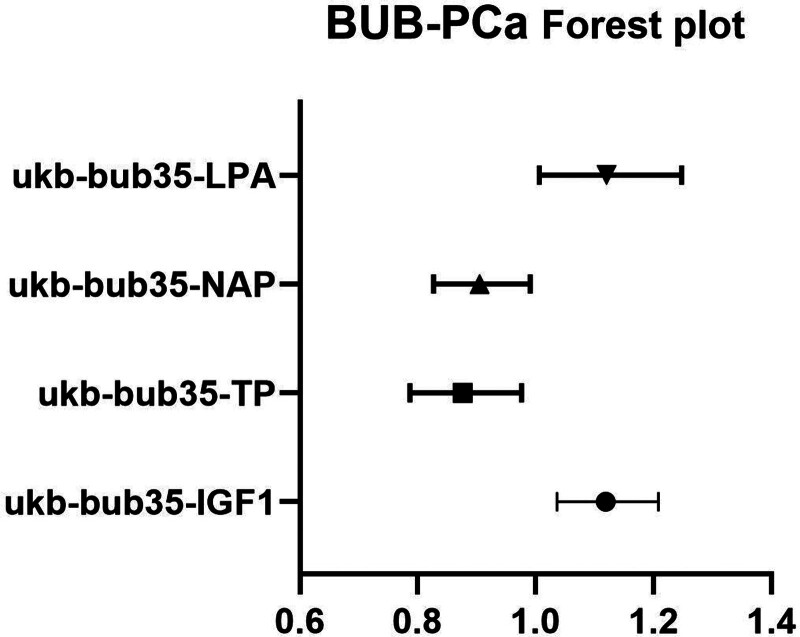
Forest plot depicting significant correlations in forward MR. The Y-axis represents diverse circulating metabolites, while the X-axis indicates OR values and corresponding 95% CIs. Various shapes in the graph symbolize distinct metabolite ORs, with the horizontal line denoting the range of the 95% CI. BUB = blood and urine biomarkers, CI = confidence interval, MR = Mendelian randomization, OR = odds ratio, PCa = prostate cancer.

The IVW analysis reveals a significant causal relation between 4 BUB and PCa (Table 6, Figs. 4 and 5). The MR analysis further indicated that UCR genetic predisposition to BPH was associated with an decreased risk of BUB, with an OR of 0.689263720210719 (95% CI: 0.463540735608078–1.02490340007659). No substantial evidence of horizontal pleiotropy among SNPs is observed (Table 7). Through MR Egger outcomes, no substantial heterogeneity is detected in relation to the association (Table 8).

**Table 6:** Forward MR IVW of BUB and PCa.

ID exposure	ID outcome	Method	NSNP	*P* value	OR
ukb-bub35-IGF1	finngen_R10_C3_PROSTATE_EXALLC	Inverse variance weighted	306	.004101067	1.119051258
ukb-bub35-TP	finngen_R10_C3_PROSTATE_EXALLC	Inverse variance weighted	216	.01657563	0.87643517
ukb-bub35-NAP	finngen_R10_C3_PROSTATE_EXALLC	Inverse variance weighted	266	.03103913	0.905333153
ukb-bub35-LPA	finngen_R10_C3_PROSTATE_EXALLC	Inverse variance weighted	13	.038093372	1.120348291

BUB = blood and urine biomarkers, IVW = inverse variance weighted, MR = Mendelian randomization, PCa = prostate cancer.

**Table 7:** Forward MR horizontal pleiotropy of BUB and PCa.

ID exposure	ID outcome	Egger_intercept	SE	*P* value
ukb-bub35-IGF1	finngen_R10_C3_PROSTATE_EXALLC	−6.30E−05	0.001711235	.970666468
ukb-bub35-TP	finngen_R10_C3_PROSTATE_EXALLC	−0.001189486	0.002128348	.576829662
ukb-bub35-NAP	finngen_R10_C3_PROSTATE_EXALLC	−0.001910363	0.001911242	.318459074
ukb-bub35-LPA	finngen_R10_C3_PROSTATE_EXALLC	0.01516834	0.009793411	.149696153

BUB = blood and urine biomarkers, MR = Mendelian randomization, PCa = prostate cancer.

**Table 8:** Forward MR heterogeneity of BUB and PCa.

ID exposure	ID outcome	Method	*Q*_pval
ukb-bub35-IGF1	finngen_R10_C3_PROSTATE_EXALLC	MR Egger	3.28E−06
ukb-bub35-TP	finngen_R10_C3_PROSTATE_EXALLC	MR Egger	7.21E−07
ukb-bub35-NAP	finngen_R10_C3_PROSTATE_EXALLC	MR Egger	8.80E−09
ukb-bub35-LPA	finngen_R10_C3_PROSTATE_EXALLC	MR Egger	0.07845393

BUB = blood and urine biomarkers, MR = Mendelian randomization, PCa = prostate cancer.

The collective findings indicate that the genetic variants associated with BUB are likely to exert a direct causal influence on the development of PCa, thereby enhancing our understanding of the potential biological pathways implicated in prostate carcinogenesis.

### 3.4. PCa reverse MR

No significant causal effect was observed between exposure to PCa and the outcome in the reverse MR analysis.

## 4. Discussion

BPH and PCa are common health concerns among males, shaped by complex interactions of biological and environmental factors. The main aim of this research is to investigate the relationship between BUB and BPH, as well as between BUB and PCa, utilizing bidirectional MR to clarify the underlying pathogenic mechanisms. This 2-way MR investigation revealed significant associations between BPH and BUB, as well as between BUB and PCa. In this study, a notable correlation was observed between BPH and GGT(*r* = 1.061658447, *P* = .028697428). PCa exhibited significant associations with insulin-like growth factor 1 (IGF-1; *r* = 1.119051258, *P* = .004101067), Lipoprotein A (LPA; *r* = 1.120348291, *P* = .038093372), TP (*r* = 0.87643517, *P* = .01657563), and non-albumin protein (NAP; *r* = 0.905333153, *P* = .03103913). Given the variability in results, the robust findings of this study indicate that alterations in GGT alone may elevate the risk of BPH. This phenomenon could be attributed to the presence of GGT, an enzyme primarily located in the liver but also present in the prostate. Elevated levels of GGT may indicate abnormal hyperplasia of prostate cells and metabolic irregularities, leading to an elevated susceptibility to prostatic hyperplasia. Interestingly, a study revealed that GGT has the potential to serve as a biomarker for PCa, as serum GGT was revealed to be elevated in PCa compared to BPH.^[22]^ There may exist a threshold for the serum level of GGT that could differentiate between BPH and PCa, necessitating additional investigation into this particular finding.

In PCa research, it has been observed that IGF-1 and LPA could potentially serve as risk factors for PCa. TP and NAP could potentially serve as protective factors for PCa. IGF-1 has been associated with PCa in prior research; however, its specificity remains limited.^[23]^ LPA is a type of lipoprotein that is inherently associated with various cancers, including PCa.^[24]^ Multiple MR studies have also uncovered a potential association between LPA and PCa.^[25,26]^ Nevertheless, further investigation is required to explore the relationship between BUB and PCa. The functions of TP and NAP may be associated with their roles in preserving physiological homeostasis and modulating immune reactions.

The findings of this study could potentially enhance the early detection of BPH and PCa from a diagnostic standpoint. Currently, the serodiagnosis of BPH and PCa primarily depends on the detection of PSA. However, the sensitivity and specificity of PSA are relatively low. The current investigation has identified a potential relationship between GGT, IGF-1, LPA, TP, and NAP in BUB with the development of BPH and PCa. Consequently, these markers could serve as novel biomarkers for the timely detection of prostate conditions. The findings may offer urologists supplementary biomarkers for the clinical diagnosis and management of BPH and PCa. These biomarkers could be linked to the prognosis of the diseases, necessitating further integration into clinical practice.

## 5. Conclusions

This study’s findings have the potential to advance the development of new therapies for BPH and PCa. Understanding the impact of GGT on the progression of prostate diseases could lead to the development of pharmaceutical interventions targeting GGT as a therapeutic approach for BPH and PCa. Similarly, by elucidating the mechanisms through which IGF-1 and LPA influence the progression of PCa, it may be feasible to devise pharmaceutical interventions that specifically target these factors for the management of PCa.

The outcomes of this study offer novel insights into the pathogenesis of BPH and PCa. Nevertheless, these findings necessitate further validation through additional experimental and clinical investigations. Furthermore, it is imperative to investigate strategies for translating these discoveries into clinical applications and utilizing blood and urine markers for the early detection and prevention of prostate ailments. Simultaneously, MR offers a novel opportunity to identify potential therapeutic targets and markers for BPH and PCa. In the future, the integration of multi-omics and big data screening, coupled with MR analysis, can be employed to identify potential aberrant targets in BPH and PCa, facilitating targeted research endeavors.

Furthermore, the utilization of extensive and robust GWAS data enhances the precision of outcomes. However, certain limitations intrinsic to this research cannot be disregarded. Notably, the generalizability of outcomes may be constrained due to the predominantly European population used in MR analysis. Subsequent research endeavors could be enriched through larger sample sizes and comprehensive metabolomic analyses, enabling a more nuanced assessment of BUB role in prostate diseases development. As our comprehension matures, the elucidation of the specific mechanisms underpinning BUB action on prostate diseases remains a pertinent avenue for exploration.

## Author contributions

**Conceptualization:** Ze-sen Lin, Min Zhu.

**Investigation:** Gui-lan Ning, Di-kuan Yang, Bao-zhong Wu, Zhi-xiong Hu, Ming-jian Liu.

**Methodology:** Gui-lan Ning, Di-kuan Yang, Bao-zhong Wu, Zhi-xiong Hu, Ming-jian Liu.

**Writing – original draft:** Ze-sen Lin, Ze-chao Zhang, Shu-ping Huang.

**Writing – review & editing:** Ze-chao Zhang, Shu-ping Huang.

## Supplementary Material
