# Supplementary material for: The association between benign and malignant prostatic hyperplastic diseases and blood and urine biomarkers: A Mendelian randomization study
Source: Medicine (Baltimore). 2025 Mar 7;104(10):e41723. doi: 10.1097/MD.0000000000041723 (PMC11903025; doi:10.1097/MD.0000000000041723)
Supplement: Supplementary file 1 [file medi-104-e41723-s001.docx]

**Forward MR**

library(TwoSampleMR)

library(ggplot2)

library(foreach)

iddf=read.table("id.txt",header =T,sep = "\t")

bioid=as.vector(iddf$myid)

result=data.frame()

foreach(i=bioid, .errorhandling = "pass") %do%{

expo_rt<-read_exposure_data(

filename = paste0("clump/",i,".txt"),

sep = "\t",

snp_col = "rsids2",

beta_col = "Effect",

se_col = "StdErr",

effect_allele_col = "ALT",

other_allele_col = "REF",

eaf_col = "MAF",

pval_col = "P-value")

expo_rt$samplesize.exposure=363228

outc_rt <- read_outcome_data(

snps = expo_rt$SNP,

filename = "myoutcome.gz",

sep = "\t",

snp_col = "rsids",

beta_col = "beta",

se_col = "sebeta",

effect_allele_col = "alt",

other_allele_col = "ref",

eaf_col = "af_alt",

pval_col = "pval")

harm_rt <- harmonise_data(

exposure_dat = expo_rt,

outcome_dat = outc_rt,action=2)

harm_rt$R2 <- (2 * (harm_rt$beta.exposure^2) * harm_rt$eaf.exposure * (1 - harm_rt$eaf.exposure)) /

(2 * (harm_rt$beta.exposure^2) * harm_rt$eaf.exposure * (1 - harm_rt$eaf.exposure) +

2 * harm_rt$samplesize.exposure*harm_rt$eaf.exposure * (1 - harm_rt$eaf.exposure) * harm_rt$se.exposure^2)

harm_rt$f <- harm_rt$R2 * (harm_rt$samplesize.exposure - 2) / (1 - harm_rt$R2)

harm_rt$meanf<- mean( harm_rt$f)

harm_rt<-harm_rt[harm_rt$f>10,]

mr_result<- mr(harm_rt)

result_or=generate_odds_ratios(mr_result)

if (mr_result$pval[3]<0.05){

result=rbind(result,cbind(id=i,pvalue=result_or$pval[3]))

dir.create(i)

filename=i

write.table(harm_rt, file =paste0(filename,"/harmonise.txt"),row.names = F,sep = "\t",quote = F)

write.table(result_or[,5:ncol(result_or)],file =paste0(filename,"/OR.txt"),row.names = F,sep = "\t",quote = F)

pleiotropy=mr_pleiotropy_test(harm_rt)

write.table(pleiotropy,file = paste0(filename,"/pleiotropy.txt"),sep = "\t",quote = F)

heterogeneity=mr_heterogeneity(harm_rt)

write.table(heterogeneity,file = paste0(filename,"/heterogeneity.txt"),sep = "\t",quote = F)

p1 <- mr_scatter_plot(mr_result, harm_rt)

ggsave(p1[[1]], file=paste0(filename,"/scatter.pdf"), width=8, height=8)

#####################################

singlesnp_res<- mr_singlesnp(harm_rt)

singlesnpOR=generate_odds_ratios(singlesnp_res)

write.table(singlesnpOR,file=paste0(filename,"/singlesnpOR.txt"),row.names = F,sep = "\t",quote = F)

p2 <- mr_forest_plot(singlesnp_res)

ggsave(p2[[1]], file=paste0(filename,"/forest.pdf"), width=8, height=8)

sen_res<- mr_leaveoneout(harm_rt)

p3 <- mr_leaveoneout_plot(sen_res)

ggsave(p3[[1]], file=paste0(filename,"/sensitivity-analysis.pdf"), width=8, height=8)

res_single <- mr_singlesnp(harm_rt)

p4 <- mr_funnel_plot(singlesnp_res)

ggsave(p4[[1]], file=paste0(filename,"/funnelplot.pdf"), width=8, height=8)

presso=run_mr_presso(harm_rt,NbDistribution = 1000)

capture.output(presso,file = paste0(filename,"/presso.txt"))

}

}

write.table(result,"result.txt",sep = "\t",quote = F,row.names = F)

**Reverse MR**

#library

library(TwoSampleMR)

library(ggplot2)

library(foreach)

iddf=read.table("id.txt",header =T,sep = "\t")

bioid=as.vector(iddf$myid)

result=data.frame()

foreach(i=bioid, .errorhandling = "pass") %do%{

expo_rt<- read.table("expo_rt_out.txt",header = T,sep = "\t")

outc_rt <- read_outcome_data(

snps = expo_rt$SNP,

filename = paste0("biodata/",i,".txt.gz"),

sep = "\t",

snp_col = "rsids2",

beta_col = "Effect",

se_col = "StdErr",

effect_allele_col = "ALT",

other_allele_col = "REF",

eaf_col = "MAF",

pval_col = "P-value")

harm_rt <- harmonise_data(

exposure_dat = expo_rt,

outcome_dat = outc_rt,action=2)

harm_rt$R2 <- (2 * (harm_rt$beta.exposure^2) * harm_rt$eaf.exposure * (1 - harm_rt$eaf.exposure)) /

(2 * (harm_rt$beta.exposure^2) * harm_rt$eaf.exposure * (1 - harm_rt$eaf.exposure) +

2 * harm_rt$samplesize.exposure*harm_rt$eaf.exposure * (1 - harm_rt$eaf.exposure) * harm_rt$se.exposure^2)

harm_rt$f <- harm_rt$R2 * (harm_rt$samplesize.exposure - 2) / (1 - harm_rt$R2)

harm_rt$meanf<- mean( harm_rt$f)

harm_rt<-harm_rt[harm_rt$f>10,]

mr_result<- mr(harm_rt)

result_or=generate_odds_ratios(mr_result)

if (mr_result$pval[3]<0.05){

result=rbind(result,cbind(id=i,pvalue=result_or$pval[3]))

dir.create(i)

filename=i

write.table(harm_rt, file =paste0(filename,"/harmonise.txt"),row.names = F,sep = "\t",quote = F)

write.table(result_or[,5:ncol(result_or)],file =paste0(filename,"/OR.txt"),row.names = F,sep = "\t",quote = F)

pleiotropy=mr_pleiotropy_test(harm_rt)

write.table(pleiotropy,file = paste0(filename,"/pleiotropy.txt"),sep = "\t",quote = F)

heterogeneity=mr_heterogeneity(harm_rt)

write.table(heterogeneity,file = paste0(filename,"/heterogeneity.txt"),sep = "\t",quote = F)

#####################################

p1 <- mr_scatter_plot(mr_result, harm_rt)

ggsave(p1[[1]], file=paste0(filename,"/scatter.pdf"), width=8, height=8)

#####################################

singlesnp_res<- mr_singlesnp(harm_rt)

singlesnpOR=generate_odds_ratios(singlesnp_res)

write.table(singlesnpOR,file=paste0(filename,"/singlesnpOR.txt"),row.names = F,sep = "\t",quote = F)

p2 <- mr_forest_plot(singlesnp_res)

ggsave(p2[[1]], file=paste0(filename,"/forest.pdf"), width=8, height=8)

sen_res<- mr_leaveoneout(harm_rt)

p3 <- mr_leaveoneout_plot(sen_res)

ggsave(p3[[1]], file=paste0(filename,"/sensitivity-analysis.pdf"), width=8, height=8)

res_single <- mr_singlesnp(harm_rt)

p4 <- mr_funnel_plot(singlesnp_res)

ggsave(p4[[1]], file=paste0(filename,"/funnelplot.pdf"), width=8, height=8)

presso=run_mr_presso(harm_rt,NbDistribution = 1000)

capture.output(presso,file = paste0(filename,"/presso.txt"))

}

}

write.table(result,"r_result.txt",sep = "\t",quote = F,row.names = F)
